# A Multikinase Inhibitor AX-0085 Blocks FGFR1 Activation to Overcomes Osimertinib Resistance in Non-Small Cell Lung Cancer

**DOI:** 10.3390/biomedicines14010066

**Published:** 2025-12-28

**Authors:** Byung-Ho Rhie, Janardhan Keshav Karapurkar, Hyun-Yi Kim, Sang Hyeon Woo, D. A. Ayush Gowda, Dong Ha Kim, Myeong Jun Choi, Young Jun Park, Viswanathaiah Matam, Yoonki Hong, Seok-Ho Hong, Suresh Ramakrishna, Kye-Seong Kim

**Affiliations:** 1 Institute of Medical Science, Kangwon National University, Chuncheon 24341, Republic of Korea; aaa0712@gmail.com; 2 Department of Internal Medicine, School of Medicine, Kangwon National University, Chuncheon 24341, Republic of Korea; h-doctor@hanmail.net (Y.H.); shhong@kangwon.ac.kr (S.-H.H.); 3 Graduate School of Biomedical Science and Engineering, Department of Biomedical Science, Hanyang University, Seoul 04763, Republic of Korea; kalpesh25021992@gmail.com (J.K.K.); tkdgus78902@naver.com (S.H.W.); ayushgowda64@gmail.com (D.A.A.G.); donha01@naver.com (D.H.K.); 4 NGeneS Inc., Ansan 15495, Republic of Korea; hykim@ngenes.co.kr; 5 Axceso Biopharma Co., Ltd., Yongin 14056, Republic of Korea; myeongjun@gmail.com (M.J.C.); yjpark@axcesobiopharma.com (Y.J.P.); 6 Department of Biomedical Science, Alliance School of Applied Engineering, Alliance University, Bengaluru 562106, India; viswanathaiah.m@alliance.edu.in; 7 College of Medicine, Hanyang University, Seoul 04763, Republic of Korea

**Keywords:** AXL, FGFR1, osimertinib resistance, AX-0085, NSCLC

## Abstract

**Background:** Osimertinib is a third-generation epidermal growth factor receptor (EGFR) tyrosine kinase inhibitor (TKI) with high efficacy in treating patients with advanced non-small cell lung cancer (NSCLC) harboring EGFR-activating mutations. Although osimertinib is a frontline anticancer agent for NSCLC, several patients inevitably develop tumor recurrence caused by osimertinib resistance. The activation of anexelekto (AXL) or fibroblast growth factor receptor 1 (FGFR1) is reported as a major factor driving osimertinib resistance in NSCLC. Thus, targeting AXL and FGFR1 offers the potential to overcome osimertinib resistance. **Methods:** In this study, we generated osimertinib-resistant cell lines from EGFR-mutant NSCLC cell lines in vitro and investigated the biological significance of AX-0085 on these cell lines by conducting transcriptomic analyses. **Results:** The expression of several genes associated with MAPK, ERK, and FGF receptor signaling pathways, including AXL, was altered upon AX-0085 treatment of osimertinib-resistant cells. Furthermore, AX-0085 treatment effectively blocked AXL and FGFR1 activation and sensitized osimertinib-resistant cells. Additionally, AX-0085 inhibited AXL and FGFR1-dependent oncogenic events, including cell proliferation, clonogenicity, and migration. **Conclusions:** The dual inhibition of AXL and FGFR1 by AX-0085 can overcome acquired osimertinib resistance, supporting its potential as a therapeutic strategy for treating patients with osimertinib-resistant tumors.

## 1. Introduction

Lung cancer is one of the most common causes of cancer-related mortality worldwide [1]. Particularly, non-small cell lung cancer (NSCLC) accounts for 85%, with adenocarcinomas comprising around 50% of NSCLC cases [1]. Extensive research on lung cancers, especially lung adenocarcinomas, showed several mutations in proto-oncogenes. Mutations in epidermal growth factor receptor (EGFR) account for nearly 50% of lung cancers in East Asians and ~15% in Caucasians [2]. Generally, EGFR tyrosine kinase inhibitors (TKIs) such as osimertinib (AZD9291), gefitinib, erlotinib, and afatinib are recommended as standard treatment for lung cancers with EGFR mutations [3].

Combinatorial targeted therapies have emerged as one of the most significant cancer treatment regimens. Osimertinib, a third-generation EGFR-TKI specifically designed to inhibit EGFR-activation mutations, has replaced first-generation EGFR-TKIs, such as gefitinib and erlotinib, as a first-line treatment for patients with metastatic EGFR mutations [4,5]. Osimertinib exhibits high anticancer activity against EGFR mutations, while only mild anticancer activity against wild type EGFR [6]. Despite the strong anticancer effects of osimertinib on lung cancers having mutations in EGFR gene contributed for acquired drug resistance [7]. Several genetic alterations are the main driving factor for acquired drug resistance to osimertinib [8,9]. In addition to genetic variations, several EGFR-dependent and EGFR-independent pathways, including EGFR C797S and T790M mutations, HER2 mutations, c-MET amplification, and epithelial-to-mesenchymal transition, facilitates to the development of acquired resistance in NSCLCs [10,11]. Hence, the discovery of therapeutic agents that reverse acquired EGFR-TKI resistance is critical for developing effective treatment strategies.

The receptor tyrosine kinase (RTK) AXL was first identified in patients with chronic myeloid leukemia [12]. The upregulation of AXL is observed in several cancers such as breast, lung, and renal cell cancer, and is linked to tumor progression with poor prognosis [13,14,15]. AXL plays a critical role in tumor growth, angiogenesis, and metastasis, including the development of resistance to anti-EGFR agents [16,17]. Overexpression of AXL has been observed in lung adenocarcinomas harboring EGFR-activating mutations, compared to those with wild-type EGFR [18]. Moreover, several studies have demonstrated that overexpression and activation of AXL signaling are associated with acquired resistance to EGFR-TKI therapies [10,17,19]. Furthermore, inhibition of AXL improves the efficacy of standard EGFR-TKI-based chemotherapy regimens [10,20].

Recently, fibroblast growth factor receptor 1 (FGFR1), another RTK, has been implicated as a mechanism of resistance to EGFR-TKIs [21]. Elevated FGFR1 expression has been linked with reduced progression-free survival in patients undergoing EGFR-TKI therapy, highlighting its potential role in diminishing treatment efficacy [22]. Upregulation of FGFR1 expression has been linked to the induction of EMT in tumor cells [23]. On activation, FGFR1 helps in recruiting fibroblast growth factor receptor substrate 2 (FRS2) adaptor protein to its juxtamembrane region. The FGFR1–FRS2 complex functions as a central hub for downstream signaling pathways critical for cell survival, including the phosphoinositide 3-kinase (PI3K)-AKT and mitogen-activated protein kinase (MAPK) pathways [24,25]. Additionally, the FGF2-FGFR1 axis, which triggers downstream PI3K/AKT and MAPK signaling, may offer an EGFR-independent survival pathway, resulting in resistance to osimertinib [26]. Given that AXL and FGFR1 share common downstream signaling molecules, it is plausible that crosstalk occurs between these two pathways, contributing to resistance mechanisms in cancer therapy. Thus, targeting both AXL and FGFR1 represents a promising strategy for overcoming resistance to osimertinib.

We have synthesized a small-molecule multikinase inhibitor, AX-0085, for inhibiting AXL activation [15]. The inhibitory efficiency of AX-0085 was significant and effectively blocked the activation of AXL in triple-negative breast cancer (TNBC). Furthermore, AX-0085 inhibited several AXL-dependent events such as cell proliferation, migration, invasion, and EMT in TNBC. AX-0085 also promoted apoptosis and cell cycle arrest by suppressing CDK2 and Cyclin E expression in TNBC. Finally, AX-0085-treated tumors displayed reduced volume in a mouse xenograft model, suggesting its potential as a therapeutic inhibitor for AXL activation in TNBC.

In this study, we aimed to further elucidate the anticancer effects of AX-0085 by examining its inhibitory efficacy on AXL and FGFR1 activation in osimertinib-resistant lung cancer cells. Transcriptome analysis revealed that AX-0085 downregulated critical genes such as FGFR1 and AXL, which were found to be elevated in osimertinib-resistant cells. Furthermore, we demonstrated that AX-0085 effectively inhibited AXL and FGFR1 activation, thereby sensitizing osimertinib-resistant HCC827 cells. Treatment with AX-0085 significantly suppressed key oncogenic processes, including proliferative capacity, clonogenic growth, and migratory activity in osimertinib-resistant cells under in vitro conditions. These findings support the potential of AX-0085 as a promising therapeutic strategy to overcome osimertinib resistance in NSCLC.

## 2. Materials and Methods

### 2.1. Cell Culture

The human NSCLC cell line HCC827, and the bronchial epithelioid cell line BEAS-2B were obtained from the American Type Culture Collection (ATCC, Manassas, VA, USA). HCC827 cells or BEAS-2B cells were maintained in RPMI-1640 medium (Gibco, Carlsbad, CA, USA), or Dulbecco’s modified Eagle’s medium (DMEM; Gibco) containing 10% FBS and 1% penicillin–streptomycin at 37 °C incubator. HCC827-osi cells were subcultured in the presence of 100 nM osimertinib. The resistant cell line HCC827-osi was established by culturing HCC827 cells containing deletions in exon 19 of EGFR with increasing concentrations (0.01–1 μM) of osimertinib.

### 2.2. Antibodies and Reagents

Antibodies were used in Western blot analysis as following: anti-hFGFR1 (Cell Signaling, #9740, Danvers, MA, USA), anti-phospho-hFGFR1 (Cell Signaling, #3476), anti-hFRS2α (Santa Cruz Biotechnology, sc-17841, Dallas, TX, USA), anti-phospho- hFRS2α (Cell Signaling, #3861), anti-hAxl (Cell Signaling, #8661), anti-phospho-hAxl (Cell Signaling, #5724), anti-Akt (Cell Signaling, #9272), anti-phospho-Akt (Cell Signaling, #9271), anti-ERK (Cell Signaling, #9102), anti-phospho-ERK (Cell Signaling, #9101), anti-PCNA (Santa Cruz Biotechnology, sc-56), anti-Ki-67 (Santa Cruz Biotechnology, sc-23900), anti-Caspase-9 (Cell Signaling, #9502), anti-Bcl-2 (Abcam, ab32124, Cambridge, MA, USA), anti-Bax (Santa Cruz Biotechnology, sc-20067), anti-Cyclin E (BD Biosciences, 554182, San Jose, CA, USA), anti-CDK2 (BD Biosciences, 610145), anti-E-cadherin (Cell Signaling, #3195), anti-vimentin (Santa Cruz Biotechnology, sc-6260), anti-N-cadherin (Santa Cruz Biotechnology, sc-393933), and anti-GAPDH (Santa Cruz Biotechnology, sc-32233). Osimertinib was purchased from Selleck Chemicals (Houston, TX, USA).

### 2.3. Western Blot Analysis

After harvesting, cells were lysed in buffer (50 mM Tris-HCl, pH 7.5; 150 mM NaCl; 1% Triton X-100; 5% glycerol; 1 mM EDTA) containing protease and phosphatase inhibitor cocktails (Roche, Mannheim, Germany). Equal amounts of protein were separated by SDS-PAGE and transferred to PVDF membranes (Merck KGaA, Darmstadt, Germany). The membranes were probed with primary antibodies at 4 °C overnight and then with appropriate secondary antibodies.

### 2.4. qRT-PCR

Total RNA was isolated and subjected to qRT-PCR as previously described [27]. Briefly, RNA was extracted using TRIzol reagent (Cat. No. FATRR001, Favorgen, Ping-Tung, Taiwan). A total of 250 ng RNA was reverse-transcribed to generate cDNA using Oligo(dT) primers (Cat. No. SO132, Thermo Scientific, Waltham, MA, USA) and SuperScript III Reverse Transcriptase (Cat. No. 18080-044, Invitrogen, Carlsbad, CA, USA). Using the SensiFAST SYBR No-ROX kit (Cat. No. BIO-98005, Bioline, GB, London), the qRT-PCR was carried out through a real-time PCR system (C1000 Thermal Cycler, Bio-Rad, Hercules, CA, USA). Primer information is provided in Table 1.

### 2.5. 3-(4, 5-Dimethyl-2-Thizolyl)-2, 5 Diphenyltetrazolium Bromide (MTT) Assay

Cell viability was measured using the MTT assay (Sigma-Aldrich, m2128, St. Louis, MO, USA). Human NSCLC cell lines were seeded into 96-well plates. The following day, cells were treated with the indicated concentrations of osimertinib or AX-0085 for 72 h. 100 µL of MTT solution (5 mg/mL in PBS) were added to the cells for 4 h at 37 °C. After incubation period, the MTT solution was taken out followed by the addition of 100 µL of DMSO to dissolve the formazan crystals. The final absorbance was measured at 540 nm using spectrophotometer.

### 2.6. Anchorage-Dependent Colony Formation

Cells were grown on 6-well plates (1 × 10^2^) cells per well. Next day, specific concentration of AX-0085 was treated on the cells. The culture medium was changed every 3–4 days, and the cells were maintained for 12–14 days. The cell culturing was stopped when visible colonies were formed. Adherent cells were stained with crystal violet (Sigma-Aldrich, Cat. No. V5265) for 30 min at room temperature. The stained colonies were visualized under a microscope.

### 2.7. Anchorage-Independent Colony Formation

Soft agar dishes were pre-coated with medium containing 0.7% agarose. Cells were grown on 6-well plates at a density of 5 × 10^3^ cells/well in 0.3% agarose onto the agar base, and the media were changed every 3–4 days. Following 12–14 days of culture, the colonies were observed under a microscope and counted.

### 2.8. Apoptosis Assay

Cells were treated with indicated concentrations of AX-0085 for 24 h. Subsequently, the cells were collected and incubated with Annexin V-FITC (BD Biosciences, Cat. No. 556547) for 20 min. Before flow cytometric analysis, propidium iodide was added, and apoptotic cells were estimated using flow cytometer.

### 2.9. Wound Healing Assay

Cells were grown on 6-well plates. Next day, the cells were subjected to the indicated concentrations of AX-0085 for 24 h. A scratch was generated using a sterile pipette tip on the cell monolayer with 80% confluence. Detached cells and debris were removed by washing with PBS. The wound closure was monitored at 0, 12, and 24 h using a Olympus IX71 inverted microscopy (Olympus, Tokyo, Japan).

### 2.10. mRNA Sequencing and Transcriptomic Analysis

The procedures for mRNA sequencing and transcriptomic analysis were performed as described previously [28].

### 2.11. Statistical Analysis

Statistical analysis and data visualization were conducted using GraphPad Prism 9.0. Data are expressed as the mean ± standard deviation from at least three independent experiments. Statistical significance was determined using Student’s *t*-test for comparisons between two groups or one-/two-way ANOVA followed by Tukey’s post hoc test for multiple group comparisons.

## 3. Results

### 3.1. Establishment of Osimertinib-Resistant EGFR-Mutant Cell Lines

Our research was initiated to understand the biological regulatory mechanisms of AX-0085 on osimertinib-resistant cells. To this end, we generated osimertinib-resistant cell lines from parental HCC827 cells, having exon 19 deletion mutations in EGFR, through stepwise exposure to increasing concentrations of osimertinib. The osimertinib-resistant HCC827 (HCC827-osi) cells showed decreased sensitivity to osimertinib treatment with a higher IC_50_ value when compared to the parental cell line (IC_50_ of parental cells = 0.01 µM; IC_50_ of HCC827-osi = 6.57 ± 0.5 µM) (Supplementary Materials Figure S1A). The effects of acquired osimertinib resistance on AKT/ERK-associated signaling pathways were assessed by Western blot analysis. The expression of phosphorylated forms of AKT and ERK was increased in HCC827-osi cells when compared with parental HCC827 cells (Supplementary Materials Figure S2A). To confirm phenotypic changes following acquisition of osimertinib resistance, we compared the expression levels of EMT (N-cadherin and vimentin) and apoptotic (Bcl-2) markers between HCC827 parental cells and HCC827-osi cells. HCC827-osi cells displayed a high expression of N-cadherin, vimentin, and Bcl-2 (Supplementary Materials Figure S2B), suggesting the occurrence of EMT in the osimertinib-resistant cell line.

### 3.2. Transcriptomic Analysis of AX-0085-Treated Osimertinib-Resistant HCC827 Cells

**Figure 1:**
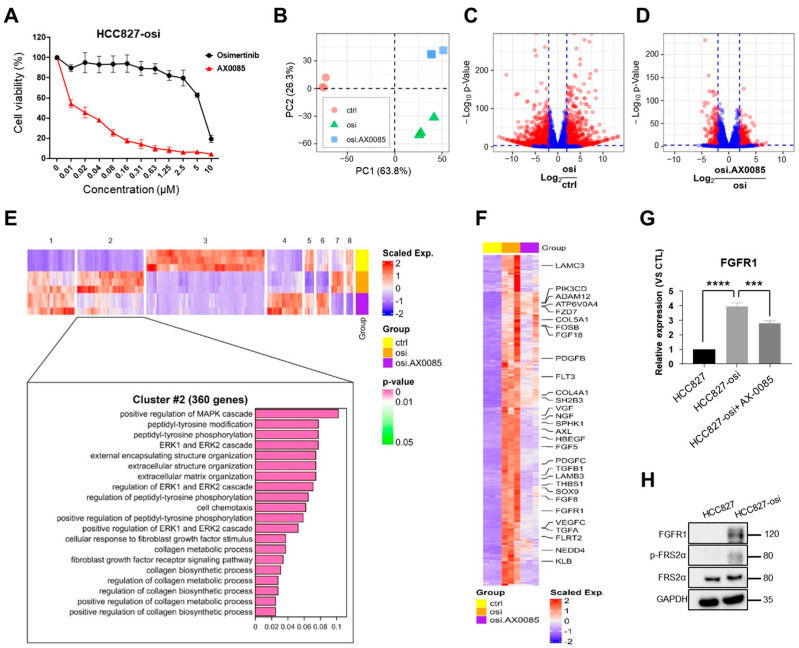
Transcriptomic analysis in osimertinib-resistant cells after AX-0085 treatment. (**A**) Osimertinib-resistant (HCC827-osi) cells were treated with osimertinib or AX0085 for 72 h. Cell viability was measured by MTT assays. (**B**) Principal component analysis was performed, and the transcriptomic distance between samples was visualized as a two-dimensional plot consisting of principal component (PC) 1 and PC2. (**C**,**D**) Differentially expressed gene (DEG) analysis was performed, and the result was visualized using a volcano plot (|Log2FC| > 2 and Benjamini–Hochberg-adjusted *p*-value < 0.001). red dots: significant; blue dots: not significant (**E**) The heatmap shows eight clusters formed by hierarchical clustering based on gene expression patterns. (**F**) cDNA microarray analysis of gene expression patterns in parental, untreated osimertinib-resistant, and AX-0085 (0.5 μM, 24 h)-treated osimertinib-resistant cells. (**G**) Verification of the FGFR1 gene, which is upregulated in osimertinib-resistant cells and downregulated by AX-0085, through qRT-PCR. Results are shown as mean ± SD based on three biological replicates (*n* = 3). Significance was assessed as follows: *** *p* < 0.001; **** *p* < 0.0001 vs. control;. (**H**) The protein expression level of FGFR1-related signaling was determined by Western blot.

Next, we assessed the effective concentration of AX-0085 (Supplementary Materials Figure S3A) on HCC827-osi cell viability [29]. Increasing the concentration of AX-0085 on HCC827-osi cells reduced cell viability accordingly (Figure 1A). To investigate how AX-0085 sensitizes HCC827-osi cells, RNA sequencing and transcriptome analysis was performed to estimate gene expression profiles across three groups: parental HCC827 (control), HCC827-osi, and HCC827-osi cells following AX-0085 treatment. We conducted principal component analysis (PCA) to visualize the transcriptomic distance among the cells, and drew a 2-dimensional plot comprising the two most informative components: principal component (PC) 1 (63.8% of the variance) and PC2 (26.3% of the variance). The distances among the groups were greater than the distances among samples within each group (Figure 1B). The samples in the parental cell group were positioned far from the samples in the HCC827-osi cell group. Furthermore, the samples in the HCC827-osi cell group were clearly separated on the basis of AX-0085 treatment, although they were relatively closer to each other than to those in the parental cell group. This indicates that the acquisition of osimertinib resistance and treatment of AX-0085 caused clear changes in gene expression in HCC827-osi cells.

To identify genes that showed significant changes by the acquisition of osimertinib resistance and the effect of AX-0085 treatment, we performed differentially expressed gene (DEG) analysis (|Log_2_FC| > 2 and Benjamini–Hochberg-adjusted *p*-value < 0.001 considered significant) and visualized the results using volcano plots (Figure 1C, D). We found 1393 significant DEGs in the HCC827-osi cells group compared to the parental cell group (Figure 1C, red dots). A comparison between the AX-0085-treated and untreated HCC827-osi cells revealed 422 significant DEGs (Figure 1D, red dots). Among these two comparisons, 1666 unique DEGs were identified, with 149 genes shared between them. We generated a heatmap that displays 8 clusters determined by hierarchical clustering based on gene expression patterns (Figure 1E).

Interestingly, among the 8 clusters identified, cluster 2, which contains 360 genes, showed a notable difference in expression pattern. A relatively higher expression of genes was found in the HCC827-osi cells compared to the parental cell group. Importantly, the high expression of genes in the HCC827-osi cell group was reduced following AX-0085 treatment (Figure 1E). Furthermore, we identified several genes within this cluster that are associated with MAPK, ERK, and FGF receptor signaling pathways, including AXL (Figure 1E,F, related genes are labeled). Thus, we investigated the inhibitory effect of AX-0085 on selected kinases. AX-0085 effectively inhibited FGFR1, AXL, and EGFR demonstrating IC_50_ values of 0.0022, 0.0044, and 0.1783, respectively, suggesting that AX-0085 had multikinase inhibitory activity (Supplementary Materials Figure S3B).

Gene ontology (GO) analysis based on genes in cluster 2 revealed top-ranked enrichment of GO terms related to the MAPK and ERK1/2 cascade (Figure 1E). A GO-gene network plot of significantly enriched signaling-related GO terms (Benjamini–Hochberg-adjusted *p*-value < 0.05) showed elevated expression of several fibroblast growth factor-related genes, including FGFR1, in HCC827-osi compared to parental cells (Supplementary Materials Figure S4A, red dots). However, most of the highly expressed genes in the HCC827-osi cell group were suppressed following AX-0085 treatment (Supplementary Materials Figure S4B, blue dots). Furthermore, we demonstrated that the mRNA expression level of FGFR1, which was increased in HCC827-osi cells, was decreased up to 1.5-fold by the treatment of AX-0085 (Figure 1G). Likewise, the FGFR1 protein level is also elevated in HCC827-osi cells (Figure 1H). Additionally, we investigated the genes which are associated with immune modulation (*CD70*, *NLRP3*, *IL6*), cell adhesion (*VCAM1*), and EMT (*ZEB1* and *TGFB1*) signaling. The AX-0085 treated HCC827-osi cells showed reduced expression of these genes, suggesting that the AX-0085 treatment on HCC827-osi cells results in reduced cell viability (Supplementary Materials Figure S4C). Thus, we aimed to assess the effect of AX-0085 on FGFR1 gene regulation and its impact on osimertinib-mediated resistance in HCC827 cells.

### 3.3. AX-0085, a Multikinase Inhibitor, Blocks FGFR1 in Osimertinib-Resistant Cells

**Figure 2:**
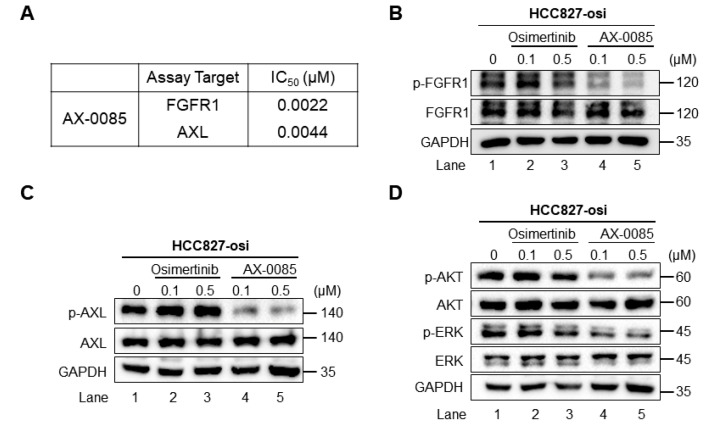
AX-0085, a multikinase inhibitor, blocks FGFR1 activation in osimertinib-resistant cells. (**A**) Inhibition of FGFR1 and AXL by AX-0085 and assessment of IC_50_ activities using cell-based kinase assay. Inhibition of the phosphorylation of FGFR1 (**B**), AXL (**C**), AKT, and ERK (**D**) after treatment with osimertinib or AX-0085 for 2 h in HCC827-osi cells.

Previously, we reported that the treatment of AX-0085 exhibited anti-tumorigenic activity by effectively blocking AXL activation in breast cancer [15]. To evaluate the inhibitory activity of AX-0085 against FGFR1, a kinase assay was performed. The results revealed that AX-0085 potently suppressed FGFR1 along with AXL kinase activity (IC_50_ for FGFR1 = 0.0022 µM and IC_50_ for AXL = 0.0044 µM) (Figure 2A). Previous reports have suggested that increased expression of FGFR1 and AXL plays a crucial role in acquired EGFR-TKI resistance by activating of the PI3K/AKT and MAPK/ERK pathways in NSCLCs [10,30,31,32]. Thus, we used the HCC827-osi cells to explore the impact of AX-0085 on FGFR1 and AXL-mediated acquired resistance to osimertinib.

The protein expression of phosphorylated FGFR1 (Figure 2B, lanes 1–3) and AXL (Figure 2C, lanes 1–3) was increased in HCC827-osi cells. However, HCC827-osi cells treated with AX-0085 displayed a significant reduction in the expression of phosphorylated FGFR1 and AXL (Figure 2B,C, lanes 4–5). Additionally, HCC827-osi cells treated with AX-0085 showed decreased phosphorylated AKT and ERK (Figure 2D, lanes 4–5), suggesting that AX-0085 effectively blocks FGFR1 and AXL activation and its downstream signaling pathways to sensitizes HCC827-osi cells by affecting cell proliferation.

### 3.4. AX-0085 Blocks the Proliferative and Colony-Forming Ability of Osimertinib-Resistant Cells

**Figure 3:**
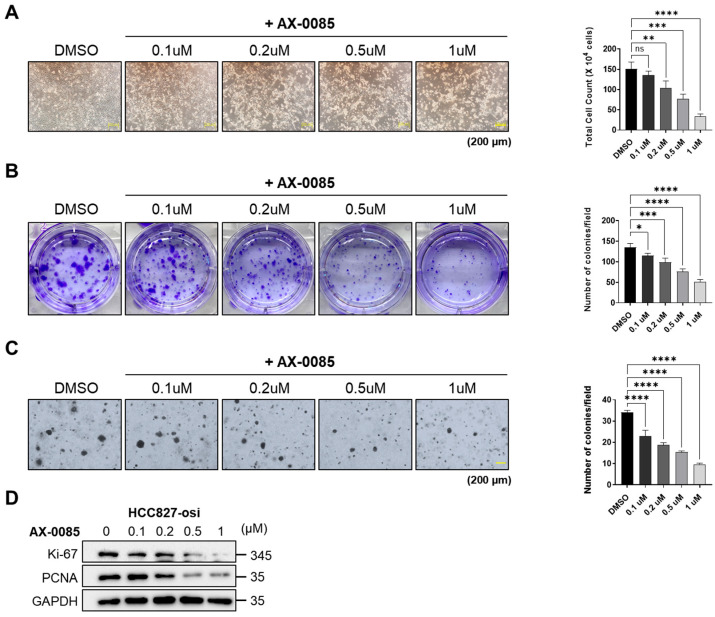
Effects of AX-0085 on the viability and colony formation of osimertinib-resistant cells. Concentration-dependent inhibitory effects of AX-0085 on the cell viability of osimertinib-resistant cells. Cells were treated with various concentrations of AX-0085 for 24 h. (**A**) Cell viability, scale bar = 200 μm, (**B**) plate colony formation of osimertinib-resistant cells, and (**C**) anchorage-independent colony formation assay, scale bar = 200 μm. Results are shown as mean ± SD based on three biological replicates (*n* = 3). Significance was assessed as follows: * *p* < 0.05; ** *p* < 0.01; *** *p* < 0.001; **** *p* < 0.0001 vs. control; ns, not significant. (**D**) Western blot analysis of Ki-67 and PCNA expression in osimertinib-resistant cells.

Cell proliferation and colony formation assays were performed in HCC827-osi cells treated with increasing concentrations of AX-0085. The cells exhibited distinct morphological alterations in a concentration-dependent manner (Figure 3A). AX-0085 treatment led to a concentration-dependent reduction in cell number (Figure 3A). Similarly, the anchorage-dependent (Figure 3B) and anchorage-independent colony-forming ability of HCC827-osi cells was decreased (Figure 3C). To obtain further insight into the intracellular signaling involved in AX-0085-mediated sensitizing of osimertinib-resistant cells, we examined the expression of proliferation-related markers following AX-0085 treatment. Cell proliferation markers, including Ki-67 and PCNA, were significantly decreased by increased concentrations of AX-0085 (Figure 3D), indicating that AX-0085 reduces the proliferative and colony-forming capacities of osimertinib-resistant cells.

### 3.5. AX-0085 Promoted Apoptosis and Suppressed the Migratory Ability of Osimertinib-Resistant Cells

**Figure 4:**
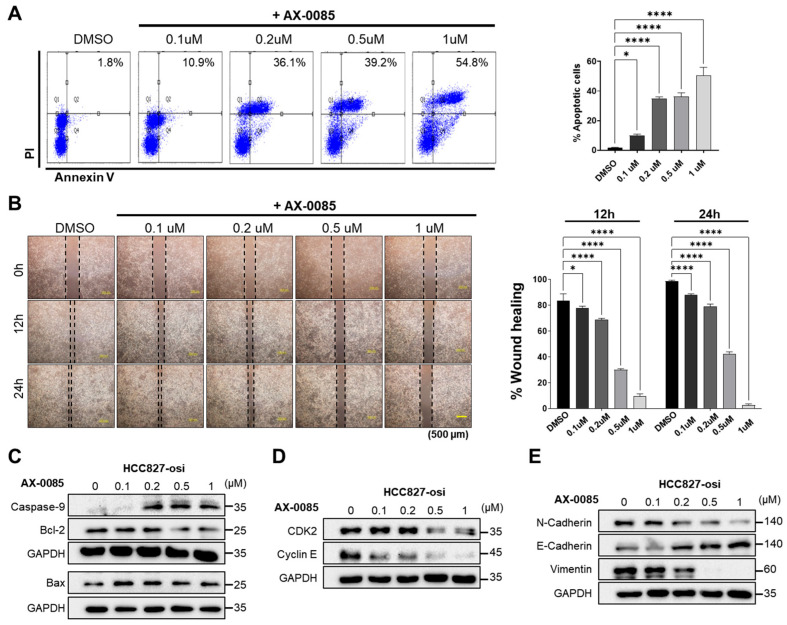
AX-0085 promoted apoptosis and suppressed the migratory ability of osimertinib-resistant cells. Osimertinib-resistant cells were incubated with AX-0085 at the indicated doses for 24 h. (**A**) Flow cytometry of cell apoptosis. (**B**) Cell migration by wound healing assays. Scar bar = 500 μm. Results are shown as mean ± SD based on three biological replicates (*n* = 3). Significance was assessed as follows: * *p* < 0.05; **** *p* < 0.0001 vs. control; (**C**) Western blot analysis of apoptosis-related proteins. (**D**) Western blot analysis of G1/S transition regulators. (**E**) Western blot analysis of epithelial and mesenchymal markers.

Next, we evaluated the anti-proliferative effects of AX-0085 in HCC827-osi cells by using an apoptosis assay. Treatment of HCC827-osi cells with AX-0085 for 24 h in an increasing concentration significantly increased the apoptotic cells (Figure 4A). We evaluated the effect of AX-0085 treatment on cell migration using a wound healing assay in HCC827-osi cells. Increasing concentrations of AX-0085 had an impact on the wound closure of osimertinib-resistant cells and prevented cell migration in a concentration-dependent manner (Figure 4B). Furthermore, cleaved caspase-9 and BAX was significantly upregulated, while the expression of the pro-survival protein Bcl-2 was downregulated in a concentration-dependent manner in HCC827-osi cells (Figure 4C), while AX-0085 did not show any toxicity on normal epithelial cells (Supplementary Materials Figure S5). Furthermore, the expression of key regulators of the G1/S transition, including Cyclin E and CDK2, was reduced (Figure 4D).

FGFR1 and AXL, members of the RTK family, play crucial roles in the EMT associated with osimertinib resistance in cancer cells [17,23]. The expression levels of the mesenchymal markers N-cadherin and vimentin were decreased following AX-0085 treatment (Figure 4E). In contrast, the epithelial marker E-cadherin showed progressively elevated expression with increasing concentrations (Figure 4E). These findings indicated that AX-0085 promotes apoptosis and cell cycle arrest in osimertinib-resistant cells.

## 4. Discussion

EGFR-targeted therapies are the first-line treatment in patients with EGFR mutations. Osimertinib, a third-generation TKI is more effective than first-generation TKIs at inhibiting EGFR with activating mutations and/or the T790M mutation. Osimertinib targets EGFR as well as non-EGFR-mediated resistance, including EMT transition, HER2 mutation, downstream activation of the PI3K or RAS pathways, and c-MET amplification [11]. Although the efficacy of osimertinib against important oncogenic drivers is recognized in patients with NSCLC, the development of acquired resistance remains a major obstacle to sustained therapeutic efficacy [33]. Recent studies have shown that the activation and upregulation of AXL and FGFR1 are linked to both acquired intrinsic resistance and bypass resistance mechanisms, leading to osimertinib tolerance [17,19,21].

FGFR1 considered as a major regulator of oncogenic signaling pathways and promote cell proliferation, survival, and differentiation in malignant cells [34]. In case of NSCLC, FGFR1 gene amplification represents the most common FGFR alteration, predominantly in squamous cell carcinoma. On contrary, FGFR1 amplification is significantly less common in lung adenocarcinoma [35]. FGFR1 amplification is mainly influenced by its complex genetic and epigenetic regulation of FGFR1 gene expression, which limits the reliability of FGFR1 gene copy number as a marker for therapeutic response. However, FGFR1 amplification is directly associated with the progression of tumors, thus pharmacological inhibition can suppress FGFR1-mediated oncogenic signaling in NSCLC [26]. Recently, several therapeutic approaches have been implemented on lung cancer models to target FGFR signaling such as small-molecule inhibitors, FGFR1-specific monoclonal antibodies and combination strategies incorporating immune checkpoint inhibitors (Supplementary Materials Table S1) [26,36,37]. Various clinical trials with AXL and FGFR1 inhibitors in patients with acquired osimertinib resistance have demonstrated limited outcomes, leading to tumor heterogeneity and drug resistance. Therefore, alternative therapeutic strategies targeting resistance to TKIs, as well as AXL and FGFR1 inhibitors, must be developed for patients with EGFR mutations [38,39].

Based on previous reports supporting the function of FGFR1 activation facilitates resistance to osimertinib, in this study, we attempted to develop an efficacious AX-0085 that targets AXL and FGFR1 tyrosine kinase [40]. To provide a model to study the efficacy of AX-0085, we generated an osimertinib-resistant HCC827 cell line harboring EGFR mutations. The osimertinib-resistant cell line showed increased resistance to osimertinib treatment compared to parental HCC827 cells. In line with previous reports [17,19,21,32], the osimertinib-resistant cell line showed increased AXL and FGFR1 expression with activation of the AKT and ERK signaling pathway (Figure 1). AKT is widely recognized as a key modulator in regulating multidrug resistance. The activation of AKT promotes cell proliferation via GSK3β phosphorylation, thereby contributing to resistance against EGFR-targeted therapy.

Activation of AXL and FGFR1 plays a crucial role in acquired resistance to TKIs, tumorigenesis, EMT, cell proliferation, invasion, and migration ability in NSCLCs [41,42]. Overexpression of AXL and FGFR1 promotes cell proliferation and migration in HCC827 cells, leading to acquired resistance to TKIs [17,21]. Notably, a number of cell processes, including cell apoptosis, proliferation, cell cycle arrest, and migration, are regulated by activation of PI3K/AKT, MEK/ERK, and MAPK pathways [43,44]. Our transcriptomic analysis revealed that the MAPK, ERK, and FGF receptor signaling pathways, including AXL, were upregulated in osimertinib-resistant cells. In contrast, osimertinib-resistant cells treated with AX-0085 reversed this upregulation (Figure 1). Furthermore, our data demonstrated that AX-0085 attenuates cellular proliferation and the ability to form colonies by inhibiting activation of AXL and FGFR1 signaling. The inhibition of AXL and FGFR1 dramatically reduced AKT and ERK activation in cells treated with AX-0085 (Figure 2). Furthermore, AX-0085 showed decreased cell proliferation and cell migration and had a reciprocal effect on apoptosis (Figure 3 and Figure 4), suggesting that the inhibitory effect of AX-0085 is partly mediated by downregulation of AXL and FGFR1-associated signaling pathways. EMT along with enhanced AXL and FGFR1 signaling plays a pivotal role in mediating acquired resistance to diverse anticancer agents, including EGFR-TKIs [17,23]. Similarly, treatment with AX-0085 suppressed EMT and decreased cell migration in osimertinib-resistant cells (Figure 4).

## 5. Conclusions

In summary, this study reports that AX-0085, a multikinase inhibitor that effectively inactivates both AXL and FGFR1, and overcomes osimertinib resistance in NSCLC. Our findings suggest that the dual inhibition of AXL and FGFR1 by AX-0085 presents a promising targeted therapeutic approach for patients with EGFR-mutant NSCLC who have developed resistance to osimertinib treatment. However, this study has certain limitations. Our data could not conclusively determine whether the reversal of osimertinib resistance is primarily driven by AXL inhibition, FGFR1 inhibition, or a combined effect of both. Since AX-0085 targets both kinases simultaneously, dissecting their individual gene functions to the observed phenotype was not feasible. In order to identify the main factor of osimertinib resistance, future studies should include AX-0085 treatment in AXL or FGFR1 gene-knocked out osimertinib resistant EGFR-mutant cell lines to address this issue.

## Figures and Tables

**Table 1:** Oligonucleotides used for qRT-PCR.

Gene	Direction	Sequence (5′ to 3′)
*FGFR1*	FP	TAATGGACTCTGTGGTGCCCTC
	RP	ATGTGTGGTTGATGCTGCCG
*GAPDH*	FP	GTCATCCCTGAGCTGAACGG
	RP	CCACCTGGTGCTCAGTGTAG

## Data Availability

The raw data supporting the conclusions of this article will be made available by the authors on request.

## Supplementary Materials

The following supporting information can be downloaded at: https://www.mdpi.com/article/10.3390/biomedicines14010066/s1. Figure S1. Establishment of osimertinib resistant cell lines; Figure S2. Characterization of osimertinib resistant cell lines; Figure S3. Chemical structure and molecular target of AX-0085; Figure S4. Transcriptomic analysis in osimertinib resistant cell after treatment of AX-0085; Figure S5. Treatment with AX-0085 did not induce toxicity in normal epithelial cells; Table S1. Representative FGFR1-targeted approaches in NSCLC.

## Abbreviations

The following abbreviations are used in this manuscript:

NSCLC	non-small cell lung cancer
RTK	receptor tyrosine kinase
EGFR	epidermal growth factor receptor
AXL	anexelekto
FGFR1	fibroblast growth factor receptor 1
FRS2	fibroblast growth factor receptor substrate 2
TKIs	tyrosine kinase inhibitors
PI3K	phosphoinositide 3-kinase
MAPK	mitogen-activated protein kinase
EMT	epithelial–mesenchymal transition
