# Supplementary material for: A Multikinase Inhibitor AX-0085 Blocks FGFR1 Activation to Overcomes Osimertinib Resistance in Non-Small Cell Lung Cancer
Source: Biomedicines. 2025 Dec 28;14(1):66. doi: 10.3390/biomedicines14010066 (PMC12838157; doi:10.3390/biomedicines14010066)
Supplement: Supplementary file 1 [file biomedicines-14-00066-s001.zip › biomedicines-4025510-supplementary.pdf]

A Multikinase Inhibitor AX-0085 Blocks FGFR1 Activation to Overcomes Osimertinib Resistance in Non-Small Cell Lung Cancer

Byung-Ho Rhie[1, 2], Janardhan Keshav Karapurkar[3], Hyun-Yi Kim[4], SangHyun Woo[3], D. A. Ayush Gowda[3], Dong Ha Kim[3], Myeong Jun Choi[5], Young Jun Park[5], Viswanathaiah Matam[6], Yoonki Hong[2], Seok-Ho Hong[2], Suresh Ramakrishna[3, 7*], Kye-Seong Kim[3, 7*]

[1] Institute of Medical Science, Kangwon National University, Chuncheon, Republic of Korea

[2] Department of Internal Medicine, School of Medicine, Kangwon National University, Chuncheon, Republic of Korea

[3] Graduate School of Biomedical Science and Engineering, Department of Biomedical Science, Hanyang University, Seoul, Republic of Korea

[4] NGeneS Inc., Ansan, Korea

[5] Axceso Biopharma Co., Ltd., Yongin, Republic of Korea

[6] Department of Biomedical Science, Alliance School of Applied Engineering, Alliance University, Bengaluru, India

[7] College of Medicine, Hanyang University, Seoul, Republic of Korea, Seoul, Republic of Korea

Running title: AX-0085 sensitizes osimertinib resistance in NSCLC

* Corresponding authors:

Suresh Ramakrishna

Address: Hanyang University, 222 Wangsimni-ro, Seongdong, Seoul 04763, Republic of Korea.

E-mail: suri28@hanyang.ac.kr; Tel: +82 2 2220 2424

Kye-Seong Kim

Address: Hanyang University, 222 Wangsimni-ro, Seongdong, Seoul 04763, Republic of Korea.

E-mail: ks66kim@hanyang.ac.kr; Tel: +82 2 2220 0601; Fax: +82 2 2220 2422

Supplementary Figures:

Figure S1. Establishment of osimertinib resistant cell lines.

Figure S2. Characterization of osimertinib resistant cell lines.

Figure S3. Chemical structure and molecular target of AX-0085.

Figure S4. Transcriptomic analysis in osimertinib resistant cell after treatment of AX-0085.

Figure S5. Treatment with AX-0085 did not induce toxicity in normal epithelial cells.

Table S1. Representative FGFR1-targeted approaches in NSCLC.

Figure S1

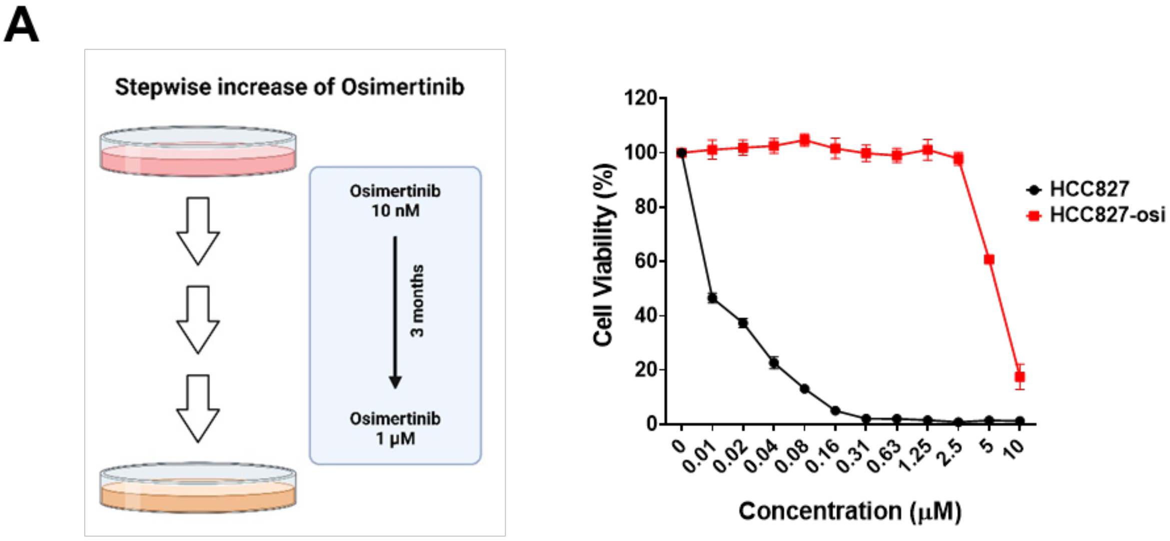

Figure S1. Establishment of osimertinib resistant cell lines.

(A) Osimertinib resistant cells (HCC827-osi) were established through continuous exposure to osimertinib using stepwise procedure (left panel). Both parental cells and osimertinib resistant cells of HCC827 were treated with osimertinib for 72hrs. Cell viability was measured by MTT assays (right panel). The IC_{50} value was calculated using GraphPad Prism software (IC_{50} of parental cells = 0.01 μM and IC_{50} of HCC827-osi = 6.57±0.5 μM)

Figure S2

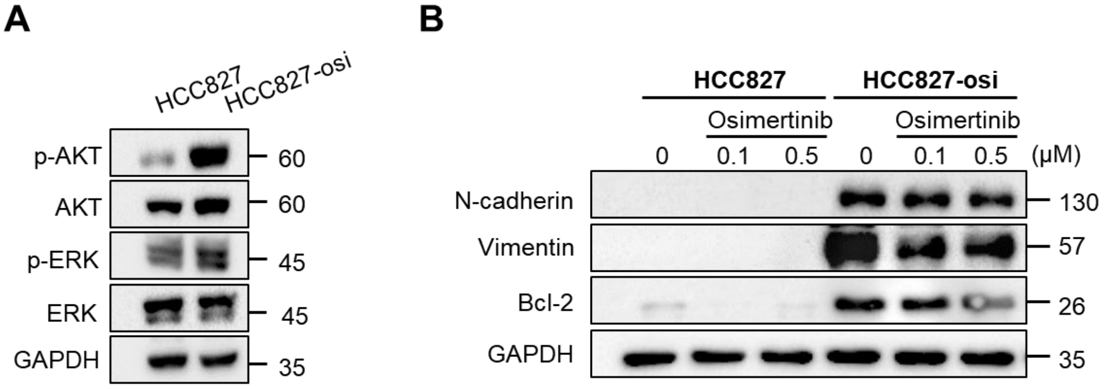

Figure S2. Characterization of osimertinib resistant cell lines.

(A) The protein expression level of AKT and ERK were determined by Western blotting. (B) The expression levels of EMT markers (N-cadherin, Vimentin) and proapoptotic marker (Bcl-2) were upregulated in osimertinib resistant cell (HCC827-osi) compared to parental cells (HCC827).

Figure S3

A

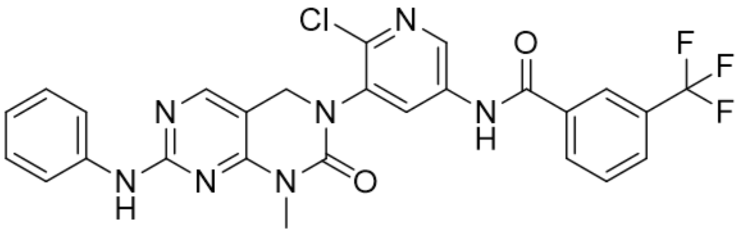

AX-0085
m.w: 553.92

B

	Assay Target	IC_{50} (μM)
AX-0085	FGFR1	0.0022
	AXL	0.0044
	ErbB1 (EGFR)	0.1783
	IGF1R	0.4385
	ErbB4	0.5226

Figure S3. Chemical structure and molecular target of AX-0085

(A) Chemical structure of AX-0085 (B) Inhibition of molecular targets by AX-0085 and assessment of IC_{50} activities using cell-based kinase assay.

Figure S4

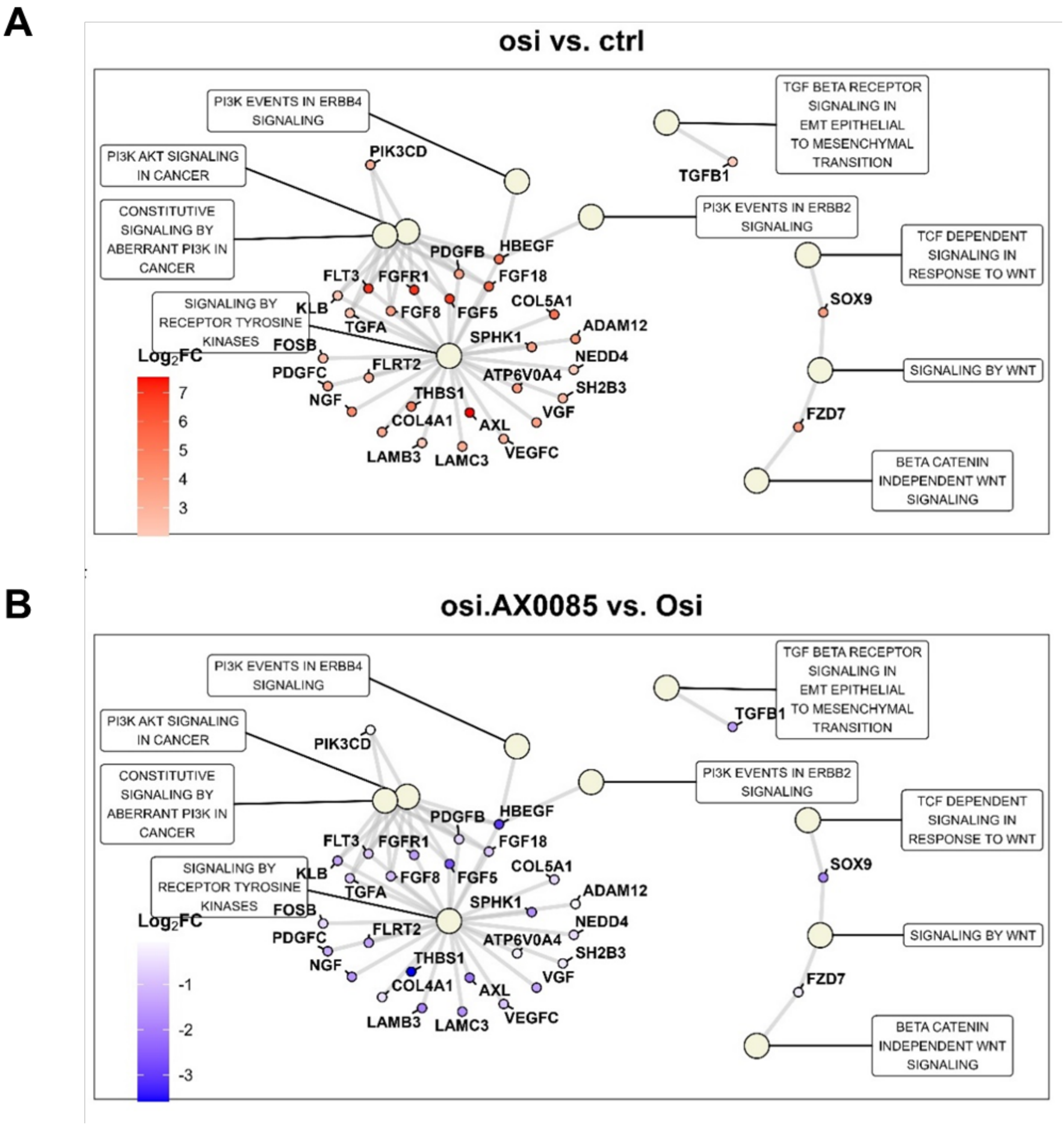
A
osi vs. ctrl
PI3K EVENTS IN ERBB4 SIGNALING
PI3K AKT SIGNALING IN CANCER
CONSTITUTIVE SIGNALING BY ABERRANT PI3K IN CANCER
SIGNALING BY RECEPTOR TYROSINE KINASES
TGF BETA RECEPTOR SIGNALING IN EMT EPITHELIAL TO MESENCHYMAL TRANSITION
PI3K EVENTS IN ERBB2 SIGNALING
TCF DEPENDENT SIGNALING IN RESPONSE TO WNT
SIGNALING BY WNT
BETA CATENIN INDEPENDENT WNT SIGNALING
PIK3CD
TGFB1
PDGFB
HBEGF
FLT3
FGFR1
FGF18
KLB
FGF8
FGF5
COL5A1
TGFA
SOX9
ADAM12
SPHK1
FOSB
NEDD4
PDGFC
FLRT2
ATP6V0A4
SH2B3
NGF
THBS1
VGF
FZD7
COL4A1
AXL
LAMB3
LAMC3
VEGFC
Log2FC
7
6
5
4
3
B
osi.AX0085 vs. Osi
PI3K EVENTS IN ERBB4 SIGNALING
PI3K AKT SIGNALING IN CANCER
CONSTITUTIVE SIGNALING BY ABERRANT PI3K IN CANCER
SIGNALING BY RECEPTOR TYROSINE KINASES
TGF BETA RECEPTOR SIGNALING IN EMT EPITHELIAL TO MESENCHYMAL TRANSITION
PI3K EVENTS IN ERBB2 SIGNALING
TCF DEPENDENT SIGNALING IN RESPONSE TO WNT
SIGNALING BY WNT
BETA CATENIN INDEPENDENT WNT SIGNALING
TGFB1
PIK3CD
PDGFB
HBEGF
FLT3
FGFR1
FGF18
KLB
FGF8
FGF5
COL5A1
TGFA
SOX9
ADAM12
SPHK1
FOSB
NEDD4
PDGFC
FLRT2
ATP6V0A4
SH2B3
NGF
THBS1
VGF
FZD7
COL4A1
AXL
LAMB3
LAMC3
VEGFC
Log2FC
-1
-2
-3

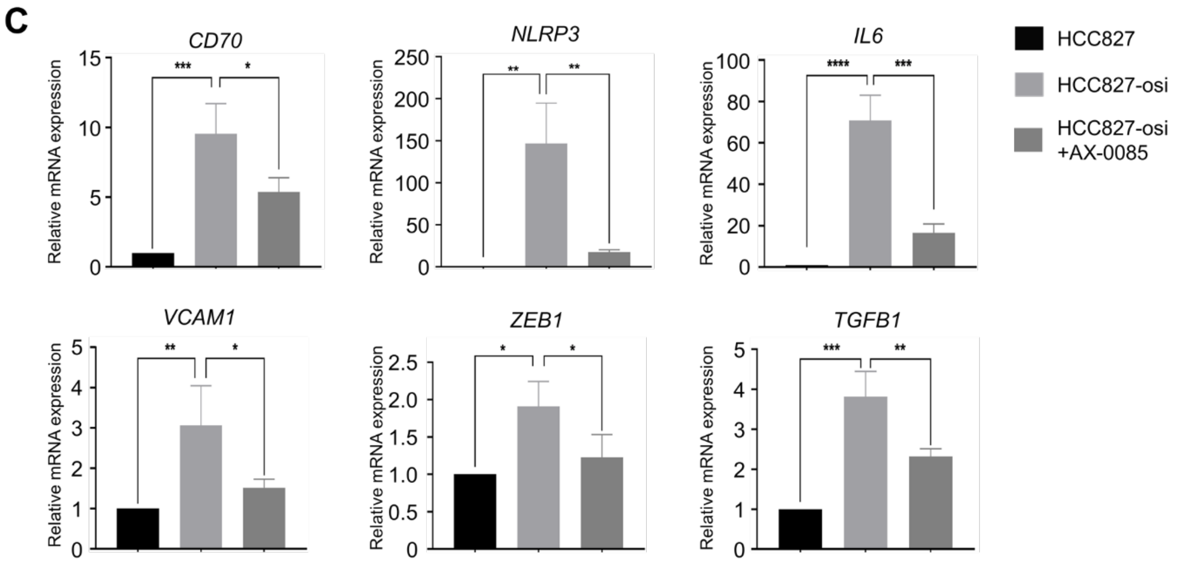

Figure S4. Transcriptomic analysis in osimertinib resistant cell after treatment of AX-0085

(A) Schematic diagram of genes which expression is upregulated in osimertinib resistant cells compared to parental cells. (B) Schematic diagram of genes which expression is downregulated in osimertinib resistant cells after treatment with AX-0085 compared to osimertinib resistant cells. (C) qRT-PCR validation on a panel of genes involved in immune modulation (*CD70, NLRP3, IL6*), cell adhesion/inflammatory signaling (*VCAM1*), epithelial–mesenchymal transition (*ZEB1, TGFB1*). Results are shown as mean ± SD based on three biological replicates (n = 3). Significance was assessed as follows: *P < 0.05; **P < 0.01; ***P < 0.001; **** P < 0.0001 vs control; ns, not significant.

Figure S5

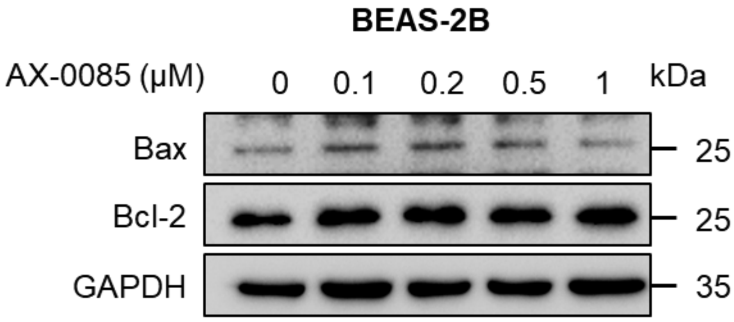

Figure S5. Treatment with AX-0085 did not induce toxicity in normal epithelial cells. Western blot analysis of apoptosis-related proteins in BEAS-2B cells.

Table S1. Representative FGFR1-targeted approaches in NSCLC

Therapeutic methods	Agents	Mechanism of FGFR1 targeting	Key findings in lung cancer	References
Small-molecule FGFR inhibitors	AZD4547 Infigratinib, Erdafitinib, Rogaratinib	ATP-competitive inhibition of FGFR1–4	Reduces FGFR1-driven proliferation, EMT, and resistance pathways; limited efficacy in unselected NSCLC	[26]
Selective FGFR1 monoclonal antibody	OM-RCA-01	Direct binding to FGFR1 extracellular domain; blocks ligand–receptor interaction	Inhibits tumor growth; enhances immune checkpoint inhibitor efficacy; significant activity in FGFR1-high NSCLC models	[36]
Multi-target FGFR inhibitors	Dovitinib, Lenvatinib	Broader inhibition of FGFR, VEGFR, PDGFR	Demonstrates anti-angiogenic and anti-proliferative effects; potential benefit in resistant tumors	[37]
Combination therapy strategies	FGFR1 blockade + (MEK or MET) inhibitor or + PD-1 inhibitors	Targeting parallel or compensatory signaling pathways	Effectively overcomes EGFR-TKI resistance mediated by FGFR1 signaling; synergistic antitumor immunity	[26,36]
